# Longitudinal Associations between Exercise and Pain in the General Population - The HUNT Pain Study

**DOI:** 10.1371/journal.pone.0065279

**Published:** 2013-06-12

**Authors:** Tormod Landmark, Pål R. Romundstad, Petter C. Borchgrevink, Stein Kaasa, Ola Dale

**Affiliations:** 1 Department of Cancer Research and Molecular Medicine, Faculty of Medicine, Norwegian University of Science and Technology, Trondheim, Norway; 2 National Competence Centre for Complex Symptom Disorders, St Olavs University Hospital, Trondheim, Norway; 3 Department of Public Health, Faculty of Medicine, Norwegian University of Science and Technology, Trondheim, Norway; 4 Department of Circulation and Medical Imaging, Faculty of Medicine, Norwegian University of Science and Technology, Trondheim, Norway; 5 Department of Oncology, St Olavs University Hospital, Trondheim, Norway; 6 Department of Anaesthesia and Emergency Medicine, St Olavs University Hospital, Trondheim, Norway; The James Cook University Hospital, United Kingdom

## Abstract

**Background:**

Population-based studies have reported conflicting findings on the relationship between physical activity and pain, and most studies reporting a relationship are cross sectional. Temporal relationships are therefore difficult to infer and associations may be subject to confounding from a variety of other factors. The aim of the current study was to investigate the association between exercise and pain longitudinally and to use within subjects analyses to remove between subjects confounding.

**Methods:**

In the population-based HUNT 3 study, participants reported both pain and level of exercise. A random sub-sample of 6419 participants was in addition invited to report their last week pain and exercise every three months over a 12 month period (five measurements in total). We used multilevel mixed effects linear regression analyses to prospectively estimate the association between regular levels of exercise (measured in HUNT 3) and subsequent longitudinal reporting of pain. We also estimated within-subjects associations (i.e. the variation in pain as a function of variation in exercise, over time, within individuals) to avoid confounding from between subject factors.

**Results:**

Among those invited to participate (N = 6419), 4219 subjects returned at least two questionnaires. Compared with subjects who reported no or light exercise, those who reported moderate levels of exercise or more at baseline, reported less pain in repeated measures over a 12 month period in analyses adjusted for age, sex,education and smoking. Adjusting for baseline level of pain distinctly attenuated the findings. Within subjects, an increase in exercise was accompanied by a concurrent reduction in intensity of pain. However, we found no indication that exercise level at one occasion was related to pain reporting three months later.

**Conclusion:**

This longitudinal population-based study indicates that exercise is associated with lower level of pain and that this association is close in time.

## Introduction

Pain complaints are common and costly. The prevalence of current pain ranges from 27% to 49% [1], [2], and the prevalence of chronic pain ranges from 11% to 64% [3]–[5]. Common pain conditions are major reasons for work related disability and for lost productivity in the work force [6], [7]. The health care expenditures among subjects with common pain complaints have been estimated to be more than twice as high as for those without pain complaints, and they seem to continue to escalate [8]–[10]. Moreover, pain is associated with a substantial reduction in self reported health and functioning [11], [12]. The best way of managing this public health problem is uncertain [13]. However, promoting a healthy lifestyle in the whole population may have beneficial effects on the prevention of pain complaints and its consequences [14].

Clinical studies have shown that exercise may relieve pain among patients with fibromyalgia and chronic low back pain [15], [16] and prevent the recurrence of low back pain after treatment [17]. However, there is conflicting evidence whether exercise relates to the occurrence of pain in the general population [18]–[21]. Results are difficult to compare due to high variability in the definitions and measurements of both activity and pain and differences in study design and population. It has been suggested that significant associations may be hidden when measures are dichotomized into active vs. inactive [22], and that physical activity may be related to the severity of pain once established [23]. In a recent study, we showed that both frequency, duration and the intensity of exercise were independently associated with a lower prevalence of chronic pain of at least moderate intensity in the general Norwegian population [24]. The cross-sectional nature of these findings limits their use in interpreting the relationship since low levels of exercise may be both a risk and a consequence of pain. Moreover, chronic pain is determined by multiple causal chains involving biological, psychological and social risk factors which may interact with or be associated with physical activity. Socioeconomic status, occupation, lifestyle and genetic makeup are factors that remain stable over time and may confound the relationship between exercise and pain. A confounder may, however, also vary across time. For example, variation in sleep, mood, or injuries may explain variations in both level of exercise and pain across time, within individuals.

In the current longitudinal population-based study, we used two separate analytical strategies to investigate the relationship between exercise and pain. First, we prospectively studied whether the baseline level of regular exercise was associated with the level of pain during 12 months of follow up. Second, we estimated the association between exercise and pain within individuals over time. When investigating the association within subjects, each individual serves as its own control and the estimates are not subject to confounding related to factors that remain stable within individuals (such as sex, socioeconomic status, occupation, genetic makeup, presence of chronic disease etc.).

## Materials and Methods

### Study Population

The basis for the present study is the Nord-Trøndelag Health Study (the HUNT study) conducted in the county of Nord-Trøndelag in Norway. The HUNT study consists of three cross-sectional surveys (HUNT 1, 1985–1987, HUNT 2, 1995–1997 and HUNT 3, 2006–2008). All inhabitants in Nord-Trøndelag aged 20 or more (N = 94194) was invited to participate in the HUNT 3 study. A total of 50839 (54%) participated. The response rate was higher among women (58%) than men (50%) and lowest among the youngest age groups (31% and 42% for the age groups 20–29 and 30–39 years, respectively). The study population is stable with sex and age distributions similar to the average of Norway, but with somewhat lower levels of education and income compared to national averages. The county is mostly rural and sparsely populated [25].

### Participants and Procedure

A random sample of 6419 HUNT 3 participants in two municipalities (Levanger and Verdal) was mailed a questionnaire and invited to participation in the current project, which main focus is on physical activity and pain. Questionnaires were mailed every three months for the following 12 months (totally five questionnaires) to those agreeing to participate (n = 4782). Reminders were mailed to non-responders together with a copy of the questionnaire after one month. If the reminder was not returned, but the subjects had not actively withdrawn from the study, no new questionnaires were mailed until the fifth mailing at 12 months follow up.

The study was approved by the Regional Committee for Medical and Health Research Ethics Central-Norway and the Norwegian Data Inspectorate.

### Questionnaire

The HUNT 3 questionnaire included three questions regarding exercise during the past year; the average number of times exercising per week *(never, less than once, once a week, 2–3 times per week or almost every day),* the average minutes each time *(less than 15 minutes, 16–30 minutes, 30–60 minutes or more than 60 minutes)* and average intensity each time (*easy, without breaking a sweat or losing breath, lose breath and brake into sweat or near exhaustion*). The questions have shown acceptable test-retest reliability with kappa values ranging from 0.52 to 0.77 and significant correlations with VO_2max_ (ranging from 0.31 for duration) to (0.43 for frequency) in adult males [26]. In a previous HUNT 3 study [24], we showed that association between frequency of exercise and prevalence of chronic pain was u-shaped among participants in working age, whereas the association between intensity of exercise and chronic pain was linear. The associations were stronger among those above working age (65 years or more) and linear in shape. To account for the unique contribution of all three dimensions (frequency, duration and intensity) of exercise, and the divergence from linearity in the association with chronic pain, we constructed a variable as follows: Those who reported no activity, light intensity activity and activity for less than 30 minutes were defined as reference group. Those reporting moderate to vigorous physical activity of 30 minutes or more were divided into two groups; those who reported 1–3 times per week, and those who reported nearly every day.

The HUNT 3 questionnaire included one question regarding pain intensity: “How much bodily pain have you had during the past four weeks?” This is a six point verbal rating scale including the response options: None, very mild, mild, moderate, severe or very severe. It has been extensively used, among others in the various versions of the SF-36 health survey [27] and is validated as a single item measure as part of the SF-8 health surveys [28].

In the one year follow up study, each of the five mailings included the one week version of the SF-8 bodily pain scale [28]. The scale was transformed according to the scoring procedures by assigning a new value to each response category based on the US SF-36 norm data [28]. This ensured a mean score close to 50 and a standard deviation close 10 in the US normative data.

Recreational exercise was defined in the follow up questionnaires by giving the following examples: going for a walk, skiing, swimming, exercise or sports. The Borg ratings of perceived exertion (RPE) scale [29] was used as an index of exercise intensity with the following instruction: “On a scale from 6 to 20, how hard is the activity that you usually do when you exercise? (Take an average from the last week). The Borg RPE scale has been shown to be a valid measure of exercise intensity in various populations [30]. In a recent investigation using the same instruction in another subsample form the HUNT 3 study, the scale corresponded well with Peak oxygen uptake (VO_2peak_) measured during an exercise test [31]. Responders were also asked how often they had engaged in recreational exercise during the last week, and the average duration each time. For the purpose of the current study, participants reporting no exercise or exercise of less than 15 minutes were assigned the value 5 and included in the Borg scale. This gave a variable ranging from 5 (no exercise) to 20 (very, very hard).

Information on the highest attained level of education was obtained from the National Education database (NUDB). Educational attainment was classified into three levels; primary, secondary and tertiary.

### Statistical Analyses

To investigate longitudinal associations between exercise and pain, multilevel mixed effects linear regression analyses were performed using the xtmixed function in Stata version 11.0 for Windows (Stata Corporation, College Station, Texas). In longitudinal studies, mixed models accounts for the dependency of observations within subjects by inducing subject specific (random) effects into the model. Missing data are handled by using all available data for each person.

First, we prospectively studied the association of exercise measured at baseline in the HUNT 3 study with the reporting of pain in the following five subsequent measurements. That is, we estimated the difference in pain during the 12 month follow up period by different levels of exercise at baseline. The estimates from these analyses were based on the mixed effects, i.e. no attempts were made at disentangling the variation within subjects from the variation between subjects. The analyses were adjusted for sex, age, education, smoking and baseline level of pain.

Second, we used the repeated measurements in the 12 month follow up to investigate within subjects associations. To disentangle the within subjects associations from the between subjects associations, two exercise variables were computed for each person: a mean score across all five measurement occasions and a deviation from the mean at each measurement occasion. The deviation scores were used to calculate the within subjects associations. These are longitudinal in that they estimate the variation in pain as a function of variation in exercise over time, within individuals. Most cross sectional and prospective analyses address research questions on a group level, such as: “Compared with individuals who report lower level of exercise, do individuals who report higher level of exercise report less pain?” Whereas within subjects analyses address questions on and individual level, such as: “Compared with time points when they report lower level of exercise, do individuals report less pain at time points when they report higher level of exercise?” In this way, subjects function as their own controls and the analyses have the advantage of not being subject to confounding by factors that remain constant over time, such as sex, socioeconomic status, genetic makeup and presence of chronic disease. In the primary model we studied whether change in exercise was associated with a simultaneous change in pain. We then investigated whether level of exercise at one occasion was associated with pain reporting three months later.

Likelihood ratio tests were used to evaluate the interactions between exercise and age and exercise and sex. Analyses were also carried out separately for each sex.

## Results

### Characteristics of the Participants

Of the 6419 subjects invited to participate in the HUNT pain study, 75% (n = 4782) responded to the baseline questionnaire (table 1). Among these, 56% were women, 28% were aged 20–44 year, 47% were 45 to 64 years and 24% were 65 years or older. One third of the participants had tertiary education, 50% had secondary education, and 17% had only primary education. Compared to the HUNT 3 population, the sex distribution was similar, whereas the proportion of middle aged and individuals with higher education were higher in the HUNT pain study. Less than 15% of the participants were lost to 12 months follow up, and attrition was neither associated with sex nor education. The proportion of subjects in the youngest age group declined somewhat throughout the follow up period. The mean pain score in the SF-8 scale (49.4; sd = 9.6) and mean exercise score on the Borg scale (11.4; sd = 3.9) were similar throughout the five occasions, indicating no attrition due to the primary study variables. Intraclass correlation coefficients (ICC) for exercise was 0.55 (95% CI 0.54, 0.57) and for pain it was 0.66 (95% CI = 0.65, 0.67). Thus, 45% of the variance in exercise and 34% of the variance in pain was accounted for by within-subject variation, respectively. This implies that the measures were quite stable, and reduces the power to detect significant within subject associations.

**Table 1:** Characteristics of the study sample at each follow up (T1–T5) and compared to the entire HUNT 3 population.

		Study sample					Hunt 3
		T1 n = 4782	T2 n = 4219	T3 n = 3926	T4 n = 3791	T5 n = 4140	n = 50827
		%	%	%	%	%	%
**Sex**							
female		56.0	56.1	56.3	56.4	56.1	54.6
male		44.0	43.9	43.7	43.6	43.9	45.4
**Age**							
20–44 yrs		28.4	26.2	25.1	24.5	26.0	29.6
45–64 yrs		47.4	48.4	48.9	49.1	49.1	43.6
65 yrs or more		24.3	25.4	26.0	26.5	24.9	26.8
**Education**							
Primary		17.2	16.9	16.7	16.8	16.7	21.2
Secondary		49.7	49.5	49.7	49.6	49.7	52.7
Tertiary		33.2	33.6	33.7	33.7	33.6	26.1

### Prospective Associations between Regular Exercise and Subsequent Pain

In the HUNT 3 study, subjects reported their level of exercise on an average week during the past year. Compared to those not reporting regular exercise in HUNT 3, those reporting at least moderate exercise 1–3 times a week reported 1.12 points less pain on the SF-8 scale (95% CI: 0.60, 1.63) during the 12 months of follow up in analyses adjusted for sex, age, education and smoking (table 2). The difference remained significant although attenuated when additionally adjusted for baseline level of pain. A similar but weaker association was seen between reports of moderate or hard exercises almost every day and subsequent level of pain. Significant interactions were seen between exercise and sex (p-value interaction<0.001). Stratified analyses revealed a stronger association between exercise of 1–3 times a week of at least moderate exercise and subsequent pain among men than women in the adjusted models (table 2). After adjustment for baseline pain the association was no longer significant for women. Significant interactions were also seen between exercise and age, revealing a stronger relationship when age increased (p-value interaction <0.001).

**Table 2:** Prospective associations between exercise* reported in the HUNT 3 study and subsequent reporting of pain† measured every third month during a 12 month follow up period of the HUNT pain study.

	Total sample		Women		Men	
	Coefficient	95% CI	Coefficient	95% CI	Coefficient	95% CI
**Unadjusted**						
None exercise	0	Ref	0	Ref	0	Ref
1–3 times/week	2.15	1.63, 2.67	1.95	1.25, 2.66	2.26	1.51, 3.00
≥4 times/week	1.53	0.69, 2.37	1.83	0.70, 2.96	1.13	−0.10, 2.36
**Adjustment** ‡						
None exercise	0	Ref	0	Ref	0	Ref
1–3 times/week	1.12	0.60, 1.63	0.78	0.66, 1.50	1.47	0.72, 2.21
≥4 times/week	0.78	0.03, 1.60	0.83	−0.28, 1.94	0.63	−0.58, 1.85
**Adjustment** §						
None exercise	0	Ref	0	Ref	0	Ref
1–3 times/week	0.42	0.23, 0.82	0.10	−0.42, 0.64	0.81	0.21, 1.40
≥4 times/week	0.32	−0.32, 0.96	0.27	−0.58, 1.11	0.34	−0.64, 1.32

^*^Average number of times per week during the last year of at least 30 minutes and either lose breath and brake into sweat or near exhaustion.

† SF-8 Bodily pain scale.

‡ Adjusted for age, education, smoking and sex as appropriate.

§ Further adjustment for baseline pain.

### Within Subjects Associations between Exercise and Pain

Within subjects associations were considered in two different temporal models (table 3). In the first model we investigated whether exercise intensity were associated with concurrently reported pain intensity (during the past week). A robust association was seen, indicating that a one point change in exercise was associated with a concurrent 0.25 point improvement in pain (95% CI: 0.21, 0.28). Thus, a 7 point increase on the scale, which would indicate a change from no to moderate exercise, would account for a simultaneous 1.75 points improvement in pain on the SF-8 scale.

**Table 3:** Within subjects associations between exercise* and pain† at the same time points (concurrent) and after three months (subsequent).

	Total sample		Women		Men	
	Coefficient	95% CI	Coefficient	95% CI	Coefficient	95% CI
Concurrent	0.25	0.21, 0.28	0.29	0.25, 0.34	0.19	0.14, 0.24
Subsequent	0.00	−0.05, 0.0)	0.01	−0.05, 0.07	−0.02	−0.08, 0.03

*Borg scale of Perceived exertion; how hard is the activity that you usually do when you exercise? (Take an average from the last week) 5 = no exercise; 20 = very, very hard.

† SF-8 Bodily pain scale.

A significant interaction was seen between exercise and sex (p<0.001). However, separate analyses revealed quite similar findings for men and women (table 3). Interaction between exercise and age (p<0.001) suggested a stronger association with increasing age.

In the second model, exercise at one occasion was not related to pain reported at a subsequent occasion (table 3).

## Discussion

In this longitudinal population-based study, regular exercise reported at baseline was associated with less pain in repeated measures over a subsequent 12 month period. However, the associations were substantially attenuated when adjusting for baseline level of pain and remained significant only for men. The within subjects analyses revealed a significant concurrent association between exercise and pain. However, no association was seen between exercise at one occasion and pain measured three months later.

Most previous population-based studies have failed to show an association between physical activity and pain [18]–[21]. It has been suggested that significant associations may be hidden when measures of physical activity are dichotomized [22] and when severity of pain is not accounted for [23]. In a recent cross sectional study we found that exercise was associated with a lower prevalence of chronic pain of at least moderate intensity, especially among older subjects [24]. In a previous HUNT study lower level of physical activity at baseline was associated with higher prevalence of widespread chronic pain 11 years later [32]. However, this study failed to account for baseline pain. It is difficult to infer any temporal relationship between activity and pain from these studies since pain might have caused reduced physical activity. One previous longitudinal study showed that physical activity was associated with less pain on the SF-36 scale measured repeatedly during three years of follow up among midlife women not reporting moderate or severe pain at baseline [33].

In the within subject analyses we found evidence for a relationship between exercise and pain that is close in time. That is, subjects reported less pain at times when they reported more exercise, whereas exercise was not related to a subsequent change in pain within individuals. This close relation in time may indicate an important reciprocity of the relationship between exercise and pain. That is, a lower level of exercise may be both a risk for and a consequence of pain. This is of particular importance when interpreting cross sectional studies of the association. However, it also shows the importance of considering baseline level of pain in prospective studies. When adjusting for baseline pain in our cohort, exercise was only related to subsequent level of pain among men. This sex difference was not as evident in the unadjusted model and may indicate that a bidirectional relationship between exercise and pain is stronger among women.

The current findings show that changes in pain might be related to exercise, in particular among men, and that the relationship is independent of time invariant factors that differ between subjects such as other lifestyle factors, sex, socioeconomic status, genetic makeup, presence of chronic disease, occupation etc.

It is difficult to draw firm conclusions about the importance of these findings. Even though we found statistically significant associations, the effect sizes were small and far from what can be regarded as clinically significant [34]. However, considering the high prevalence of chronic pain [5], even low effect sizes could have public health significance. That is, if we could increase the level of physical activity in the population, chronic pain could potentially be prevented in a noticeable number of subjects. Future studies should use long term follow up with the aim at identifying the proportion of cases with significant chronic pain that might be prevented by regular exercise. Moreover, the relationship is likely to be stronger in certain clinical populations than in the population at large [15], [16]. Identifying subgroups that may benefit more from exercise interventions on a population level may therefore be an objective for future investigations.

Some considerations regarding the statistical analyses need to be mentioned. When modelling within subjects associations, the factors of interest must vary within individuals. In the current study both pain and exercise were relatively stable. This may have reduced our power to study longitudinal associations as only those individuals with time related variations contributed to the within subject estimates. Still, the number of participants was substantial and the model was able to detect significant relations. Although these analyses removed the confounding of time invariant factors, factors that may vary within individuals, such as injuries, mood, sleep and anxiety could have confounded the associations. However, these factors may be part of causal chains between physical activity and pain, and including them as time-varying covariates in the analysis would require quite complex theoretical models of the relationships [35]. We assumed a liner relationship after having plotted the SF-8 pain scores against the exercise scores in a cross sectional dataset. A linear association between intensity of exercise and prevalence of chronic pain was also reported in a previous cross sectional study [24]. In that study, the frequency of exercise was not linearly associated with the prevalence of chronic pain, however, and this was also evident in our prospective analyses which indicated a weaker association with pain as the frequency of exercise exceeded 3 times a week. In the prospective analyses we adjusted for baseline pain. In some cases, when there is considerably measurement error, adjustment for baseline scores of the outcome variable might cause inflation of the association [36]. Such adjustments should therefore be done with caution. Our adjustments, on the other hand, led to an attenuation of the associations, which was in accordance to what would be expected.

In the time lag model, we did not find evidence for an association between exercise during one week and subsequent changes in pain. One possible explanation for this might be that the three month intervals between measurements were too long. That is, exercise during one week might have been related to pain during the next week, although it was not related to pain during one week three months later. However, the lack of evidence for an association in the time lag model corresponds with the attenuation of the estimates in the prospective analyses after adjustments for baseline pain, indicating that only a limited change in pain was seen over the one year course as a function of regular exercise reported at baseline.

We had to rely on self report measures. In terms of pain there is no alternative as pain per definition is a subjective experience. Even though the verbal rating scale we used to assess pain is well validated [28], it is unlikely to possess ratio qualities, i.e. equal intervals between the categories. Nevertheless, it has been increasingly recognised that parametric statistics, such as regression analyses, are valid for ordinal pain scales, at least those containing 5 categories or more [37]. Objective measures of physical fitness are likely to give more valid results than self reports of physical activity [38], [39]. However, the repetition of measurements at five occasions during one year in a large population-based sample would require extensive financial resources and even though the activity may change, measures if fitness would not change in the same degree. We therefore used the Borg Scale of perceived exertion which gives detailed information on exercise intensity. The scale is well validated and, self-reports of usual exercise intensity is independently associated with VO_2peak_ in the general population [31].

### Conclusion

This longitudinal population-based study gives robust evidence for an association between exercise and pain. However, the association was close in time and weak, and its importance remains open to debate.
